# Clinical Applications of Adipose-Derived Stem Cell (ADSC) Exosomes in Tissue Regeneration

**DOI:** 10.3390/ijms25115916

**Published:** 2024-05-29

**Authors:** Konstantinos S. Papadopoulos, Christina Piperi, Penelope Korkolopoulou

**Affiliations:** 1 Department of Plastic and Reconstructive Surgery, 401 General Military Hospital of Athens, 11525 Athens, Greece; dean.papadopoulos66@gmail.com; 2 First Department of Pathology, Medical School, National and Kapodistrian University of Athens, 11527 Athens, Greece; 3 Department of Biological Chemistry, Medical School, National and Kapodistrian University of Athens, 75 M. Asias Street, 11527 Athens, Greece

**Keywords:** adipose-derived stem cells, exosomes, regenerative medicine, wound healing, musculoskeletal regeneration, tissue engineering

## Abstract

Adipose-derived stem cells (ADSCs) are mesenchymal stem cells with a great potential for self-renewal and differentiation. Exosomes derived from ADSCs (ADSC-exos) can imitate their functions, carrying cargoes of bioactive molecules that may affect specific cellular targets and signaling processes. Recent evidence has shown that ADSC-exos can mediate tissue regeneration through the regulation of the inflammatory response, enhancement of cell proliferation, and induction of angiogenesis. At the same time, they may promote wound healing as well as the remodeling of the extracellular matrix. In combination with scaffolds, they present the future of cell-free therapies and promising adjuncts to reconstructive surgery with diverse tissue-specific functions and minimal adverse effects. In this review, we address the main characteristics and functional properties of ADSC-exos in tissue regeneration and explore their most recent clinical application in wound healing, musculoskeletal regeneration, dermatology, and plastic surgery as well as in tissue engineering.

## 1. Introduction

In the last few years, regenerative medicine has seen remarkable advances due to the therapeutic properties of stem cell-based therapies, with applications in the fields of oncology and tissue engineering. Among several stem cell types, adipose tissue-derived stem cells (ADSCs) are considered most promising due to their abundance and high yield from fat tissue that can be achieved by simpler, effortless isolation and less invasive methods than bone marrow extraction. Moreover, ADSCs’ ability to differentiate into multiple cell types enables their multipotency and suitability for several regenerative applications. Their regenerative properties are mediated by the secretion of several growth factors and cytokines, which induce angiogenesis and tissue repair. ADSCs also exert immunomodulatory properties, with low immunogenicity that enables allogeneic transplantation and reduces the risk of immune rejection. They are highly proliferative cells that can be easily cultured in vitro at a low cost, maintaining genetic stability over long periods of time, and being effectively used in diverse tissue regeneration approaches without raising any ethical issues due to their from adult tissue [1].

However, beyond the conventional paradigm of cell regeneration, an innovative avenue of research has gained new momentum, focusing on the role of extracellular vesicles, and especially exosomes, secreted by ADSCs (ADSC-exos). These membrane vesicles have attracted interest because they can mediate intercellular communication and target cells both locally but also in a systemic context [2]. Although the stem cells’ regenerative potential has been extensively investigated, the molecular signaling network orchestrated by their exosomes presents a new and complex dimension with immense therapeutic approaches that require extensive investigation.

Encouraging scientific evidence comes from cancer studies where exosomes have been attributed a multidimensional role in the progression and metastasis of tumors through the transport of bioactive compounds that can modulate the tumor microenvironment, also offering the biological vehicles of targeted drug delivery. At the same time, their application in tissue engineering assures impressive results in the development of advanced regenerative therapies. Their ability to mediate the healing mechanism, stimulate wound neovascularization, and regulate inflammation points towards their central role in creating a favorable environment for tissue regeneration and neo-organ construction. By investigating the complex mechanisms through which these exosomes exert their effects on recipient cells, current research data are gradually paving the way for innovative regeneration strategies. In this review, we discuss the characteristics and functional properties of ADSCs followed by an update on the isolation methods and advances of ADSC-exos in tissue regeneration, and their potential significance in clinical settings.

## 2. Characterization and Isolation Methods of the Multipotent ADSCs

ADSCs present a class of multipotent mesenchymal stem cells (MSCs) which have the capacity of self-renewal and differentiation into other cell lineages. Their potential to release a wide range of active biological molecules has enabled their use in novel, promising approaches to regenerative medicine treatments. The International Society for Cellular Therapy has defined the MSCs as adherent cells that show plasticity and express CD90, CD105, and CD73 cell surface markers under standard culture conditions while being negative for CD11b, CD14, CD19, CD34, CD45, CD79a, and HLA-DR, with ability to differentiate into adipocytes, chondroblasts, and osteoblasts in vitro [3].

Studies unraveling the secretome of MSCs from different origins, such as adipose tissue, placenta, bone marrow, or Wharton’s jelly, have detected some differences during proteomic analysis depending on the cell origin [4]. However, there was no clear superiority in the functional analysis of the secretome since both cell migration and resistance to cell death signals were found equally enhanced [5]. The autologous use of somatic cells is preferred, with adipose tissue being a generous place of the origin of MSCs. Moreover, mesenchymal cells isolated from adipose tissue have been up to 500 times more than those derived from the bone marrow, while the areas abundant in stem cells are the subcutaneous abdominal, visceral, and omental fat [6].

ADSCs exhibit discrete morphological and immunophenotypic characteristics, appearing mostly as fibroblasts and expressing CD13, CD29, CD49d, CD73, CD90, and CD133 markers as well as MHC I/II. However, they do not express CD106 which is present in bone marrow stem cells (BMSCs) [7]. Furthermore, ADSCs possess the same multipotency as BMSCs, but they are isolated in an easier way, in larger numbers, and less invasively [8]. The use of autologous ADSCs is common in clinical practice because these cells retain their ability for prolonged survival and differentiation while they also have numerous applications in wound healing, lipotransfer for aesthetic or reconstructive reasons, and osteochondral regeneration [9]. 

ADSCs are easily harvested, mainly from the subcutaneous tissues of the anterior abdominal wall, with techniques such as direct fat excision, with classic liposuction, or the Coleman suction method, with the latter two being superior in the percentage of viable cells (Table 1) [10]. These methods can be further combined with centrifugation, sonication, or enzymatic tissue digestion techniques (collagenase, 0.25% trypsin, trypsin-EDTA, etc.) for the final isolation of a purified fracture [11]. 

Enzymatic cell dissociation is inferior to mechanical techniques when comparing the number of viable ADSCs, as they manage to isolate an increased number of cells with greater colony-forming potential and an increased expression of transcription factors (e.g., Nanog and Oct4) that exhibit a decisive role in maintaining the multipotent nature of the cells along with their ability to differentiate [12]. However, the disadvantage is that the enzymatic procedures require the use of xenogeneic products and must adhere to strict research protocols; nevertheless, there are also allogeneic products that have been developed, such as Liberase™, a mixture of xeno-free proteases, which is shown to be equally effective in isolating ADSCs in relation to commonly used enzymes [13]. Recently, hybrid tissue digestion methods were studied in patients and revealed that a specific mechanical separation to obtain an ADSC-rich material called stromal vascular fraction (SVF) may represent a superior method in the preservation and survival of fat grafts, requiring further validation [14].

SVF can also be derived from adipose tissue and in addition to mesenchymal adipocytes, it is rich in fibroblasts, macrophages, lymphocytes as well as smooth muscle and endothelial cells. A high percentage of cells (85%) in SVF express CD34, along with CD13, CD73, CD90, and CD105 markers while being negative for CD45, CD235a, and CD31. Some studies have demonstrated that cultured ADSCs are CD34- [15] which exhibit a greater capacity for osteochondral differentiation [16].

However, SVF is quite heterogeneous in composition and therefore does not present the most suitable material for allogeneic use, but it exhibits increased angiogenic activity, and a greater proliferative and colony-forming potential compared to ADSCs [17]. The use of fat autografts has been investigated in a study employing SVF as a cell-assisted lipotransfer with statistically significant fat graft survival and reduced complications (e.g., fibrosis and cyst formation) [18].

ADSCs and SVFs can be stored for future application by using a variety of media with various components (e.g., human serum and serum albumin) with cryopreservation (−70 °C) for up to two years, without a reduction in their population, but with comparatively reduced viability, colony formation, and survival [19]. Subsequently, these cells can be cultured and used in the future for regenerative purposes or in bio-scaffolds for tissue engineering, aiming for the ex vivo growth of tissues and organs [20]. 

ADSCs are also suitable as allogeneic cell therapies because they exhibit low immunogenicity as a result of the low expression of major histocompatibility complex II (MHC II) molecules and the in vitro production of CD80, CD86, and CD40 markers by T and B cells [21]. However, ADSCs cannot be considered as immunologically inactive cells since they can elicit both a humoral and a cellular immune response in vivo. Moreover, they can be affected by inflammation and induce MHC I/II molecule expression as well as Toll-like receptors [22,23]. In vitro cell cultures may also alter the immunological properties of adipose stem cells, which can be overcome to a small extent with the addition of fetal bovine serum (FBS), a common cell culture media supplement which slightly reduces cellular immunogenicity [24].

## 3. Functional Characteristics of ADSCs Based on Their Origin

Subcutaneous and visceral adipose tissue exhibit distinct characteristics and functional properties in metabolic regulation. For instance, visceral fat exerts a stronger inflammatory reaction than subcutaneous fat. These differences are possibly attributed to their embryological origin as the subcutaneous fat derives from the somatic lateral plate mesoderm while the visceral fat develops from the visceral lateral plate mesoderm. ADSCs obtained from visceral or subcutaneous tissue do not show significant differences in regenerative potential, while obtaining them from subcutaneous tissue is considerably superior as it is quick, easy, and less invasive compared to visceral tissue [25].

However, RNA-sequencing analysis showed different transcriptional characteristics between the two types of mesenchymal cells, visceral and subcutaneous ADSCs. ADSCs from both tissues may show diverse effects considering the mechanisms of fibrosis, adipogenesis, angiogenesis, or the induction of inflammation and present heterogeneous gene expression profiles. Visceral mesenchymal cells have a more adipogenic and pro-inflammatory effect, while subcutaneous mesenchymal cells show similarities with their progenitor cells and exhibit anti-inflammatory properties.

Both types of mesenchymal cells express the same surface markers and similar cell viability in transplantation but differentiate in several functions such as motility, adhesion, cytokine secretion, and gene expression, which are affected by the microenvironment. Compared to visceral ADSCs, subcutaneous ADSCs can easily differentiate into adipocytes and osteoblasts, but the former release high inflammatory cytokines levels, including IL-6, IL-8, and TNF-α [26].

A study has demonstrated the characterization of three different subtypes of human progenitor adipocytes, grouped according to their respective CD34 expression (CD34−, CD34low, CD34high). The ADSCs of visceral adipose tissue have a similar number of CD34− and CD34hi cells, whereas in the adipose tissue of the abdominal subcutaneous fat, ADSCs are mostly CD34−. From other studies, the subcutaneous ADSCs are characterized by CD10 expression, while those of visceral fat express mainly CD200 [27]. The adipogenic ability is associated with CD10+ and CD200- cells, which is consistent with the high differentiation of subcutaneous than visceral ADSCs, in response to adipogenic stimuli [28]. On the other hand, the latter express reduced CD90 markers and proliferate more slowly, demanding stronger signals to differentiate and being associated with metabolic disorders. This could hypothetically justify the hypertrophy of the existing adipocytes of visceral fat in response to obesity. In contrast, subcutaneous ADSCs express high levels of CD90 with a concomitant enhanced proliferation, mitotic activity, and adipogenic differentiation [29].

Two categories of adipose tissue with different physiology and functions have been identified, brown (BAT) and white adipose tissue (WAT). BAT is formed in early developmental stages, being mostly present in newborns [30]. In adults, it is present in the supraclavicular region, neck, axilla, and paraspinal regions, correlating with body mass index (BMI) [31]. Brown adipocytes have large numbers of mitochondria and fat droplets, they show sympathetic innervation and express the UCP-1 protein which is involved in heat generation through the respiratory chain, while additionally showing resistance to oxidative stress due to mitochondrial biogenesis and the continuous oxidation of fatty acids during thermogenic stimulation [32]. Brown fat develops from the paraxial mesoderm (dermomyotome) as in muscle and dorsal dermis. The expression of the transcription factor Ebf2 presents specificity for BAT and their precursor cells [33]. WAT can be converted to BAT upon exposure to cold or other sympathetic stimuli (e.g., β3-adrenergic signals) through the expression of Ebf2, which signals and controls the molecular profile of brown pre-adipocytes [34]. 

BAT-derived MSCs (BAT-MSCs) can be distinguished from WAT-derived stem cells with respect to their origin and cell lineage properties. Specifically, WAT-MSCs are derived from Myf5 (myogenic regulatory factor 5)-negative lineages, while BAT-MSCs exhibit Myf5 expression and are derived from myogenic differentiation lineages. The BAT-MSCs can be grown in vitro, exhibiting an active metabolism and multilineage differentiation properties, capable to differentiate into brown adipocytes. When compared to WAT-MSCs, their gene expression profile was associated with *PGC-1a*, *PRDM16*, *CREB1*, and *UCP1*, capable to induce the osteogenesis, adipogenesis, and chondrogenesis of brown and white adipocytes [35]. Following these differences, exosomes derived from BAT-ADSCs and WAT-ADSCs exhibit differences in their molecular composition and functional properties. BAT-ADSC-Exos contain miRNAs, lipids, and proteins involved in thermogenesis and mitochondrial function such as UCP-1, miR-133, and miR-155 while WAT-ADSC-Exos carry miRNAs and proteins involved in adipogenesis, extracellular matrix remodeling, and the regulation of inflammation as well as signaling lipids involved in insulin sensitivity and the modulation of inflammation. These molecular differences give the ability to BAT-ADSC-Exos to be involved in metabolic regulation aspects related to thermogenesis and mitochondrial function, being therefore applied in obesity treatments or muscle regeneration and the repair of ischemic tissues while WAT-ADSC-Exos can affect cell proliferation, adipocyte function, inflammation, and insulin sensitivity and can be used for type 2 diabetes applications as well as bone regeneration, wound healing, and soft tissue repair [36,37].

## 4. Isolation Methods of ADSC-Exosomes

Multiple methods have been described in the literature for the isolation of exosomes with differential ultracentrifugation being one of the most commonly used methods for exosome isolation [38]. The combination with techniques like ultrafiltration and dichotomic size-exclusion chromatography can amplify the purity by decreasing possible nano-debris and preserve the functional cluster of the isolated exosomes [39]. 

Every scientist seeks specific features when isolating exosomes for their research, such as high purity and the recovery of large amounts of exosomes, increased activity, or fast and cost-effective methods. Commercial kits for specifically isolating MSC-exos are widely available at the moment and relatively cheap but they use precipitation and fluorescent labeling for the high recovery of low-purity isolated EVs. A new certified in vitro diagnostic test (CE-IVD) has been recently available to assess the risk for prostate cancer in the liquid biopsies of the urine [40]. Also, the harvested exosomes from commercial kits can be enriched with polyethylene glycol (PEG) to rapidly isolate large quantities for analysis. However, these PEGylated exosomes may exhibit altered effects in clinical use, since they are immunologically active and lead to anti-PEG IgM synthesis by T-cells that affect their blood clearance rates [41].

Traditional methods may be adequate for studies that do not require detailed omic-analysis but their heterogenous results are a major disadvantage [42]. To this end, modern methods have been developed in order to maximize efficiency depending on the specific aims of the study. For example, microfluidic-based exosome isolation is a high-purity low-cost method that functions on chip-like devices while magnetic technologies and microarrays work via specific binding for high purity and fast results. Some of the latter methods are also ideal for the clinical setting and can be used for liquid biopsies, but their downside resides in the limited availability and the high cost [43,44,45].

## 5. Bioactive Cargos of ADSC-Exos and Factors Affecting Their Production

ADSC-exos contain a variety of bioactive cargos including proteins such as growth factors transforming growth factor-beta (TGF-β), hepatocyte growth factor (HGF), and vascular endothelial growth factor (VEGF) promoting angiogenesis and tissue repair, as well as anti-inflammatory cytokines involved in immune response and inflammation control and matrix metalloproteinases implicated in tissue remodeling. Exosomal membrane lipids are involved in structural integrity but also contribute to cell signaling and promote survival and uptake by recipient cells. Apart from these bioactive lipid molecules, ADSC-exos also contain nucleic acids such as miRNAs and lncRNAs which control gene expression post-transcriptionally, and mRNAs that can be translated to functional proteins and induce several cellular functions in recipient cells. The DNA fragments of genomic and mitochondrial origin are also present in exosomes with a potential modulating role in the function of recipient cells [36,37].

The biomolecular profile of exosomes has been shown to be affected by the culturing conditions and priming of MSCs modulating both their content as well as their function. In more detail, culturing conditions such as low oxygen induce the production of exosomes with enhanced regenerative properties along with the secretion of growth factors that enable angiogenesis and tissue repair. Serum and nutrient presence or absence from culture media may also change the RNA and protein content of exosomes. Moreover, scaffolds or three-dimensional (3D) cultures can also alter their properties compared to 2D monolayers.

In addition, the priming of MSCs with inflammatory cytokines (IL-6, TNF-α, etc.) or growth factors (TGF-β and HGF) can change the composition of exosomal cargos, modifying protein levels or enzymes as well as lipid composition. The exposure of MSCs to stress conditions (such as oxidative stress, hypoxia, or heat-shock) can affect the miRNAs and proteins present in exosomes, further enhancing angiogenesis and tissue repair. 

Zhao et al. demonstrated, through two consecutive studies in experimental animals and humans, that ADSCs cause the M2 polarization of macrophages with simultaneous “beiging/briting” of WAT with the exosomes of ADSCs presenting the key mediators in the intercellular interactions of these two cell types [46,47]. M2 macrophages display regenerative and immunoprotective properties and usually appear in the later stages of inflammation, following interleukin (IL-4, IL-13, and IL-10) and TGF-β signals. They are mainly involved in tissue healing, ECM deposition, and angiogenesis but they also suppress the immune response and further activation of T-cells through the secretion of anti-inflammatory cytokines [48]. 

On the contrary, M1 macrophages are activated types of macrophages, stimulated by pro-inflammatory cytokines (IFN-γ) and bacterial products such as lipopolysaccharides in the early stages of inflammation and tissue injury, when a strong inflammatory response is required to eliminate pathogens and start the healing process. Moreover, M1 macrophages secrete pro-inflammatory cytokines (IL-1, IL-6, and TNF-α) that recruit other cells and enhance the immune response. In addition, they display antigen presentation and microbicidal activity through the formation of nitric oxide (NO) and reactive oxygen radicals (ROS) [49].

The anti-inflammatory and immunosuppressive activity of mesenchymal cells has been confirmed in many studies [50] and is most likely attributed to the factors of the inflammatory environment, rather than to an indigenous trait of the cells themselves [51]. As mentioned above, pre-activated ADSCs exposed to pro-inflammatory cytokines (TNF-α and IFN-γ) show significant changes in several miRNAs of their exosomes. As a result, the amplification of miR-34 and miR-146 in ADSC-exos induced the polarization switch of M1 macrophages to M2 through interaction with the monocytes of the microenvironment, thus regulating gene expression and chemokines that suppress the inflammatory reaction, such as prostaglandin E2 (PGE2), Indoleamine 2,3-Dioxygenase 1 (IDO), IL-8, and IL-10 [52].

ADSCs also respond to the mechanical stimuli of the microenvironment in a specific way. Chandler et al. studied the response of ADSCs to mechanical stimuli in the presence of cancer and showed that these cells, like cancer cells, contract and grow faster in a compact environment, where forces of ~10 kPa are exerted, through ROCK kinase-induced changes in the cytoskeleton. They subsequently observed the increased production of growth signals such as VEGF and IL-8 and the inhibition of the adipogenic differentiation of ADSCs, with the coexistence of tumor cells and ADSCs leading to greater local tumor infiltration and desmoplasia [53].

## 6. Clinical Applications of ADSC-Exosomes

Exosomes, being the extracellular vesicles of small caliber that are essential for communication between cells, have been involved in numerous molecular pathways and recent studies highlight their crucial role in cell proliferation, differentiation, and signaling regarding oncogenesis, migration, and metastasis as well as in the regeneration of tissues [54]. In the following sections, we explore their most recent clinical applications in wound healing, musculoskeletal regeneration, dermatology, and plastic surgery as well as in tissue engineering.

### 6.1. ADSC-Exos in Wound Healing

As previously described, an important function of mesenchymal cells, and subsequently of their exosomes, is their immunomodulatory effects on macrophages, which have a primary role in tissue regeneration utilizing their unique ability to rapidly change phenotype and functional characteristics [55]. Exosome-based therapy can regulate the behavior of neighboring cells and promote tissue healing and regeneration, retaining the benefits of acellular therapies. It is therefore anticipated to be an additional tool in the clinicians’ armamentarium for treating acute and chronic wounds along with other modern means including hyperbaric oxygen, negative pressure wound therapy (NPWT), and other biological therapies.

The healing process is inextricably linked to the adequate blood perfusion of the tissues. Angiogenesis is an innate ability of the body to ensure optimal perfusion during the early steps of healing, being mediated by angiogenic factors, including FGF, TGF, and VEGF-A. Exosomes play an important role in this pathway, with cargoes like miR-30b that facilitate angiogenesis, enhancing the production of pro-angiomiRs by HUVECs as well as transducing growth signals towards angiogenesis and increased blood flow [56]. Exosomes and EVs have been found to promote healing through specific cargoes such as miR-486-5p, which was also enriched in ADSC-EVs co-cultured with human microvascular endothelial cells (HMECs) and human dermal fibroblasts (HDFs), enhancing full-thickness wound healing through neoangiogenesis [57]. 

Other studies demonstrated that ADSC-exos transfer miR-125a to endothelial cells to induce angiogenesis [58], whereas upon culturing in endothelial differentiation media (EDM), they enhance angiogenesis by transferring miR-31 to endothelial cells [59]. 

In addition, the lncRNA MALAT1 is present in human ADSC-exos, presenting a factor that, when used locally in the wound, stimulates healing by inducing HDF proliferation and migration, with a mechanism similar to FGF-2 [60]. Another pathway affected by MALAT1 ADSC-exos is Wnt/β-catenin signaling axis, which is associated with multiple functions such as migration, cell proliferation, epithelialization, and ECM remodeling. Exosomes with MALAT1 appear to induce the Wnt/β-catenin pathway via miR-124 binding in vitro and ultimately inhibit oxidative stress in HDF and HaCaT keratinocytes incubated in H_2_O_2_ [61].

Diabetes mellitus represents the greatest challenge to the healing mechanism in clinical practice due to the disruption of tissue perfusion by microangiopathy but also due to many other complex metabolic disorders. The positive effects of exosomes are presumably unaffected by diabetes as shown in studies with experimental diabetic animal models, increasing neo-vessels in the early phase and collagen fibers in the later phases of healing. This has been attributed to the ability of ADSC-exos to infiltrate fibroblasts and stimulate their proliferation [62]. It is reported that ADSC-exos can simultaneously stimulate endothelial progenitor cells (EPCs), which degenerate in high glucose levels, promote new vessel formation, and express antioxidant transcription factors such as Nrf2, which plays a central mediator role in cell survival under the conditions of oxidative stress [63]. Consequently, this increases granulation tissue formation and growth factor expression levels, as well as angiogenesis while reducing inflammation and oxidative stress levels in the diabetic wound through a mechanism similar to that of hyperbaric oxygen treatments [64].

Regardless of the angiogenic potential, ADSC-exos can transfer their cargo to the aforementioned human dermal fibroblasts and stimulate their growth and migration towards the wound bed. These molecules promote the conversion of TGF-β3 to TGF-β1, the production of collagen III and metalloproteinase MMP-3 via the ERK/MAPK pathway, and ultimately the remodeling of the ECM to optimize healing without scar formation [65]. This study used intravenous ADSC-exos and showed that the amount of granulation tissue and the conversion of fibroblasts to myofibroblasts decreased, which paradoxically means that the trauma will have less contraction, and therefore an increased chance of creating a scar. However, the study has monitored them indirectly via α-SMA myofibroblasts on days 14 and 21 after exosome treatment, and it is possible that these cells may have already undergone apoptosis in the setting of optimal healing, indicating that the mechanism by which exosomes act needs further investigation and carefully designed experimental models [66].

The existence of cells such as fibroblasts and keratinocytes in the wound is another key parameter for better and complete healing. ADSC-exos may induce fibroblast growth and migration, regulating collagen deposition by activating the PI3K/Akt signaling pathway and subsequently accelerating wound healing in mice with skin defects [67]. More specifically, it appears that this pathway is induced by ADSC-exos rich in miR-21 that cause the proliferation of keratinocytes (HaCaT cells) and the simultaneous increase in MMP-9 [68]. 

Additionally, MSC-exos from three different sources, including stem adipocytes, were shown to secret FGF-2, VEGF-A, HGF, and PDGF-BB. Through these factors, they were able to positively affect wound healing through keratinocyte migration, the remodeling of ECM, and fibroblast differentiation into myofibroblasts [69]. These exosomes were further shown to express transmembrane proteins and some intracellular proteins, such as argonaute protein 2 (AGO2), CD63, and CD9, known exosomal markers. A particularly interesting feature of this study is that the cells were incubated in xeno-free media, which makes this protocol directly applicable to clinical studies. It is worth mentioning that AGO2, also reported in several other studies, exhibits a critical role in the guidance and binding of microRNA to its mRNA target, and is perhaps one of the key mechanisms of the selective action of exosome cargoes, which deserves further study [70].

### 6.2. Musculoskeletal Regeneration through ADSC-Exos

**Figure 1:**
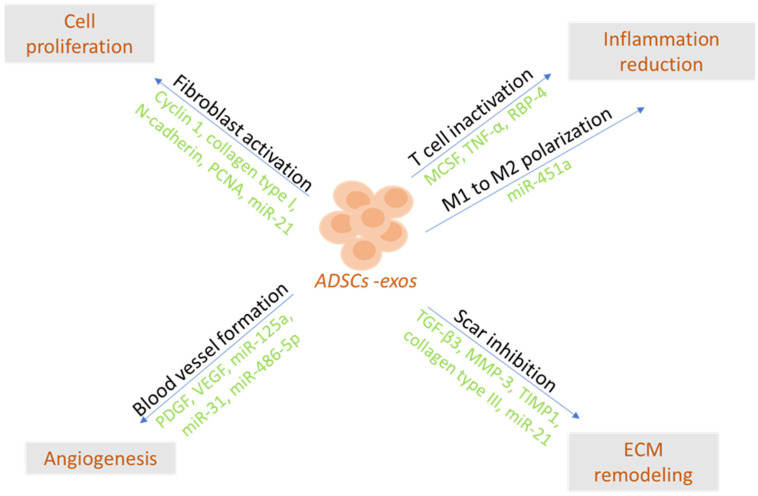
ADSC-Exos may accelerate healing by reducing early inflammation, inducing angiogenesis and cell proliferation, and modulating extracellular matrix remodeling [83].

ADSCs promote muscle regeneration through the induction of signals from various signaling pathways and specifically through their exosomes and EVs, even without the mandatory presence of the cells themselves. The study of Mitchell et al. used ADSCs, the fraction of EVs, and the total secretion of these cells in multiple sequential experiments, and their results highlighted the distinct role that each of these cell derivatives has in specific functions depending on the environment [71]. The total secretion of ADSCs appeared to possess the most regulatory potential in muscle regeneration, angiogenic ability, and the inhibition of cell degeneration by modifying the microenvironment through several different microRNAs. This observation was expected, since the total secretome contains a plethora of growth factors, cytokines, and immunoregulatory molecules relative to the fraction of EVs (Figure 1). Nevertheless, ADSC-EVs contained equally important miRNAs, such as two strong pro-angiogenic factors, miR-23a and miR-23b, and the miR-let7 family that drives macrophages to M2 polarization which, as mentioned above, has an anti-inflammatory role, while miR-24, miR-125b, and miR-16 induced a negative regulation to MYD88/NF-κB, IL-6/TNF-α, and IKK-α, respectively. Overall, this series of events led to the reduction in fibrosis in chronic inflammation [72,73]. 

In other aspects of the skeletal system, ADSC-exos have been shown to use their specific cargoes to promote chondrogenesis from periosteal cells in vitro through miR-145 and miR-221 [74], to induce the osteogenic differentiation of ADSCs themselves via miR-130a-3p and the SIRT7/Wnt/β-catenin pathway [75] as well as after incubation in osteogenesis-inducing media with an increase in Runx2 and ALP [76]. In addition, molecularly modified exosomes from ATDC5 cells (chondroprogenitor cell-derived exos) can also be enriched with VEGF plasmids to promote osteogenesis in bone defects by enhancing angiogenesis [77]. Another factor that appears to positively affect the chondrogenic capacity of ADSC-exos is the small bioactive molecule, kartogenin (KGN). KGN-induced ADSC-exos have been shown to induce the growth and migration of ADSCs as well as their chondrogenic differentiation. They were also found to upregulate the expression of the genes that are implicated in chondrogenesis such as *collagen II/III*, *Aggrecan*, and *SOX9* and significantly decrease the expression of the genes that are involved in chondrolysis such as *ADAMTS4*, *MMP-3*, and *ADAMTS5* [78].

Bone regeneration through ADSC-exosomes or other mesenchymal cells has a great dynamic when combined with scaffolds, aiming to induce new bone production in situ. The techniques and candidate materials that have been used are discussed in detail below; nevertheless, there is evidence for the importance of exosomes in bone regeneration.

Exosomes from 3D cultured ADSCs, when administered intravenously to mice with large cranial bone defects, appeared to have greater pro-osteogenic capacity than simple cell cultures [79]. In addition, ADSC-exos appear to act protectively against corticosteroid-induced bone apoptosis and oxidative stress through Nrf2 expression in dexamethasone-exposed osteoblasts and thereby restore bone loss [80].

In other applications, adipogenic capacity was correlated with miR-450a-5p, as EVs from adipose tissue can induce ADSCs to differentiate into mature adipose tissue via the inhibition of WISP2, a sub-pathway of the Wnt pathway [81].

ADSC-exos can also be internalized by Schwann cells and induce proliferation, myelination, and nerve regeneration in peripheral nerve lesions through the overexpression of specific mRNAs, such as MMP-9 [82].

Hypoxic conditions were shown to promote the growth of a robust regenerative fraction of EVs. A study of a muscle degeneration model showed that EVs derived from MSCs cultured under hypoxic conditions were more effective in inducing muscle regeneration than EVs from normoxic cultured MSCs [84]. There is evidence that MSCs present a more unstable secretome, which depends on many factors, and some studies suggest that although the EV fraction of MSCs supports muscle regeneration, the specification of the putatively effective factors is highly variable.

Moreover, the paracrine functions of MSCs were attributed to growth factors and cytokines, as well as other factors, such as exosomes. Therefore, MSC-derived exosomes may act as a key regulatory factor contributing to skeletal muscle regeneration. In addition, ADSC-exos contain significantly more functional microRNAs, one of which is the anti-apoptotic miR-21 that promotes endothelial cells towards angiogenesis by enhancing Akt and ERK signaling pathways and upregulating hypoxia-inducible factor 1 (HIF-1α) and stromal cell-derived factor 1 (SDF-1) expression [85]. Another study showed that miR-1, miR-133, and miR-206 have myogenic activity while miR-494 strongly promotes myogenesis alongside angiogenesis. These miRs, although independent of common myogenic cytokines such as FGF-2, GCSF, and PDGF-BB, act in a similar paracrine manner and lead to the upregulation of factors such as VEGF and IL-6 [86]. 

The mechanism of action and effects of ADSC-exos in different target cells are summarized in Table 2.

A comparative study of bone marrow mesenchymal cell exosomes and adipose tissue-derived exosomes in the efficacy of acellular osteoarthritis treatment showed the former being slightly superior in producing new type II collagen [87]. Nevertheless, ADSCs are more easily accessible, easier to harvest, and collected in greater numbers. Moreover, they are not required to be cultured in the case of auto-transplantation and it is therefore suggested that the efficacy of ADSCs in clinical practice is superior.

### 6.3. ADSC-Exos in Dermatology and Plastic Surgery

The clinical use of exosomes is slowly starting to emerge for different clinical applications because of the greater safety characteristics of acellular therapies. In the field of dermatology and aesthetic medicine, treatment with ADSC-exos has already been tested at the clinical level, although these applications are off-label and further studies are demanded to validate their safety and efficacy.

ADSC-exos have the ability to protect HDFs and HaCaTs from UVB radiation and promote collagenogenesis and the production of antioxidant elements through increased TGF-β expression while mitigating ECM remodeling through reduced MMP-1 and IL-6, which ultimately leads to greater skin elasticity and protection against photoaging [88]. In the same context, keloid fibroblasts grown with ADSC-exos exhibited reduced activity and a higher probability of apoptosis through the suppression of the TGF-β1/SMAD pathway [89]. In this way, it is suggested that ADSC-exos seem to exert more of a regulatory role in maintaining normal homeostasis in the regenerative cycle than acting as the exclusive stimulators or repressors of molecular pathways.

In other studies, ADSC-exos were found to improve the symptoms of atopic dermatitis by reducing the production of inflammatory cytokines such as IL-4, IL-5, IL-13, IFN-γ, and TNF-α [90] and promoting hair and follicle maturation and regeneration in vivo [91].

Exosomes also have clinical applications in autologous fat transplantation. Autologous fat grafting, or lipotransfer, is employed extensively in plastic surgery in various parts of the body for aesthetic or reconstructive reasons. The grafted tissue is most commonly obtained using a liposuction cannula and subsequently undergoes enzymatic or mechanical processing in order to collect the SVF with the maximum concentration of ADSCs [92].

ADSC-exos were co-incubated with umbilical vein cells and were shown to enhance the maintenance of fat graft volume and quality through neoangiogenesis [93]. This indicates that HUVECs and SVF cells potentially play an important role in inducing mesenchymal cell exosomes to behave as angiogenic factors, possibly through pro-angiogenic miRNAs such as miR-30b [32,74]. Hypoxic cell culture media are perhaps the keystone to graft survival because ADSC-exos derived from cells incubated in such media may have even greater potential to attract HUVECs and promote fat graft survival via neoangiogenesis and the regulation of VEGF/VEGFR [94,95].

A modern and promising contribution of ADSC-exos to reconstructive plastic surgery concerns the improvement in perfusion and survival of flaps. A flap refers to a technique in which a type of tissue (skin, subcutaneous tissue, fascia, or bone) is collected from a donor site and transferred to another recipient site maintaining random or specific vascularization, without, however, the requirement of neoangiogenesis (e.g., free tissue grafts). The importance of flaps lies in the fact that their perfusion confers enhanced strength and survivability in contrast to an avascular graft, with the ultimate goal of covering extended traumatic or oncological defects that would otherwise fail to heal [96]. However, a flap can often face problems with its endogenous vasculature, either due to the patient’s vascular pathology (diabetes mellitus, atherosclerotic disease, etc.) or due to poor surgical technique with a risk of ischemia [97]. The ADSCs grown in media with H_2_O_2_ (H_2_O_2_ preconditioned ADSCs) produced exosomes that contributed to the survival of the flaps undergoing ischemia/reperfusion injury (I/R) [98] while the ADSC-exos that stimulate the expression of IL-6 in the neoangiogenesis of the flaps with I/R were shown to have a similar effect [99].

## 7. ADSC-Exo Applications in Tissue Engineering

Tissue engineering is an interdisciplinary field that employs biological principles with medicine and engineering to generate functional tissues and organs in a laboratory setting. Its ultimate goal is tissue repair or the replacement of dysfunctional or lost tissue in the human body to achieve structure and function as close to normal as possible.

### 7.1. Scaffolds and In Vitro Tissue Engineering Approaches

The key elements of tissue engineering include the usage of scaffolds, donor cells that can be cultured and used to colonize the scaffold, and finally proliferation signals such as growth factors, cytokines, or other signaling molecules that are used to modulate cell behavior, promote specific cell differentiation, and enhance the integration of the engineered tissue. Living cells are isolated from a donor source, ideally homologous, and are cultured in the laboratory. These cells may be stem cells or specialized cells derived from the target tissue. These cells are “seeded” on the scaffold, where they adhere, multiply, and differentiate to form the desired tissue. Scaffolds can be biodegradable or non-degradable but should be biocompatible, as a framework to support cell growth and tissue formation in vivo. Cell sheets are a class of scaffolds, which can be enriched with mesenchymal adipocytes to act as an adjunct in full-thickness wounds and promote healing [100]. Recently, numerous different materials can serve as scaffolds in experimental settings that are coupled with large amounts of exosome-laden scaffolds, combining the advantages of acellular therapies through active biomaterials that possess structural and biological integrity. These materials aim to continuously release exosomes in the target area or organ, preventing their phagocytosis by macrophages and prolonging their time of action [7].

The scaffold ought to provide a three-dimensional structure that resembles the ECM of the target tissue and guides the organization of the cells in space. Three-dimensional (3D) scaffolds with specific pore diameters have been found to be the optimal environment for cell growth, as they supply a larger surface area for better cell adhesion and migration, mimic normal tissue with respect to plasticity and mechanical stimuli, and trigger neoangiogenesis in vivo. For example, chondrocytes had optimal growth on polycaprolactone (PCL) scaffolds with 400 μm diameter pores [101]. 

Another interesting example involves the alginic acid (Alg) hydrogels. ADSC-exos have been used in conjunction with alginate hydrogel scaffolds in wound healing models to improve significantly collagen synthesis, healing, and the formation of new vessels in the wound area. In addition, Alg hydrogel’s physical and biochemical features have been studied along with ADSC-exos and it was shown to be fully biodegradable and biocompatible [102].

Autologous cellular scaffolds based on collagen and platelet-rich plasma (PRP) and enriched with ADSC-exos have been shown to promote healing through the effect on keratinocytes and fibroblasts [103]. Cellular scaffolds may show slightly inferior biocompatibility compared to collagen scaffolds but offer a clear advantage in the proliferation potential of human periosteal cells in a comparative study performed with Bio-Gide^®^ and PRF (platelet-rich fibrin) scaffolds [104]. PRF is easily reproduced from autologous peripheral blood collection and centrifugation, has the desired three-dimensional shape, and can be a key component for composite scaffolds that need to be investigated in future studies.

Osteogenic scaffolds using MSC-exos have also been proposed for acellular osteochondral regeneration. Recently, many selective scaffolds have been created, such as porous 3D scaffolds made of polylactic acid (PLA) coated with MSC-exos, and were studied for their immunoregulatory and osteogenic abilities. The porous 3D-PLA-exo scaffold was studied and found to possess immunoregulatory properties, mainly by reducing the pro-inflammatory reaction and toxic metabolites (ROS), exhibiting osteogenic abilities when BMSCs were cultured in it. As a result, the cells expressed higher mRNA levels of ALP and the osteogenic transcription factor Runx2, which is crucial for MSC differentiation into osteoprogenitor cells and ultimately into osteoblasts [105].

In the study of Kim et al., 3D porous silk fibroin (SF) scaffolds were created and demonstrated that human ADSC-exos can induce in vitro bone differentiation and regeneration in human BMSCs and in an animal model with bone defects [106].

Similar studies in different scaffolds observed the same osteogenic activity towards BMSCs. The materials of these scaffolds included hydrogels with ADSC-exos, enriched with miR-375, which promotes bone production [107], or more solid materials such as PLGA (poly-lactic-co-glycolic acid) enriched with ADSC-exos that were produced under osteogenic stimuli and aimed to be released steadily in the area of the bone defect until complete healing [108]. 

Polyethylene glycol (PEG) hydrogels are another category of synthetic polymers that are biocompatible and can be easily manipulated to create scaffolds. A customized injectable PEGylated hydrogel has been described to carry the functional mRNAs of VEGF-A and BMP-2 from small EVs and other miRNAs, generated by human ADSCs after their infection by engineered plasmids carrying the same factors [109]. 

Another way to transport ADSC-exos to target cells is the use of gelatin nanoparticles (GNPs). Gelatin nanoparticles are nanoscale particles made of gelatin, a natural biopolymer derived from collagen, characterized as biocompatible, biodegradable, and non-toxic. Experimental animal studies treated with miR-451a-loaded ADSC-exos and GNPs showed increased bone regeneration in cranial bone defects due to the immunomodulatory effect of M2 polarized macrophages [110]. Also, gelatin combined with polydopamine, a biomimetic polymer, was used to create a gelatin sponge and was enhanced with ADSC-exos in experimental models with BMSCs in vitro and with bone defects in vivo. These results were, also, relevant to the osteogenic capacity of exosomes, showing increased mRNAs of genes related to osteogenesis and guiding the differentiation of BMCs, while osteogenesis was confirmed by the imaging and histological analysis of the in vivo experimental models [111]. 

In reference to other examples, a 3D-printed gelatin methacrylate scaffold, which mimics ECM, was enriched with exosomes and induced the M2 polarization of macrophages, enhanced chondrocyte migration, and restored dysfunctional chondrocytes in an osteoarthritis model [112]. 

The ability for the M2 polarization of macrophages was also maintained by porous polyethylamine scaffolds combined with polycaprolactone (PCL) fibers and enriched with MSC-exos (exo-PEF scaffolds), which could sustain a maximum volume of exosomes up to 30 μg, which were maintained on the material until they came into contact and interacted with cells. These properties arise when the PCL fibers undergo electrospinning so that when they are directed to form three-dimensional structures with very fine nanoscale fibers and pores. Controls in scaffolds without exosomes tended to attract CD86+ M1 polarized macrophages, in contrast to exo-PEF scaffolds, which favored CD206+ M2 polarized macrophages and CD4+ T-regulatory cells and their combination led to the ideal immune response for promoting tissue regeneration [113].

Conversely, 3D-printed titanium nanoparticles combined with macrophage-derived exosomes, containing BMP2 (bone morphogenetic protein 2) and ALP, were studied and shown to be able to guide autologous mesenchymal cells to bone differentiation [114].

### 7.2. In Vivo Tissue Engineering Approaches

In vivo tissue engineering involves the process of manipulating or modifying biological systems within a living organism. It may require the introduction of new genetic material, cellular components, or other entities such as biocompatible materials, into an organism to achieve a specific therapeutic effect, for example, tissue regeneration or the fabrication of new tissue by combining exogenous agents with native cells to repair complex tissue defects.

One of these techniques is the creation of prefabricated flaps. Flaps, as mentioned above, are often used to repair or replace damaged or missing tissue, improve blood circulation in the absence of adequate healing, cover exposed structures, and even create new organs and parts of the body (e.g., reconstruction of the mandible with the fibula, neophallus creation from forearm skin) with compound flaps composed of a combination of skin, fascia, muscle, fat, and bone [115,116].

The first study of prefabricated flaps described a two-step procedure that initially involved the insertion of a vascular pedicle into a tissue segment with the desired characteristics and the transfer of the tissue complex with its vascular pedicle to the recipient area after neoangiogenesis [117]. A subsequent study redefined prefabricated flaps by using free tissue grafts in areas delineated by specific vascular pedicles, thereby adding new and unique tissue layers to flaps, a technique named prelamination [118].

In recent years, significant advances have been made in the research of skin substitutes or other types of tissue that have been constructed in vivo with the help of living, vascularized tissues in the context of regenerative and reconstructive surgery. Some of the commonly used adjuncts for this purpose are cell sheets of various materials, homologous or allogeneic acellular tissues, and tissue engineering chambers (TECs) [119]. TECs are used to mimic specific conditions or create a controlled isolated environment for the evaluation of potential treatments.

Many researchers are combining these methods, for example, creating prefabricated tissue segments with autonomic perfusion by fusing autologous tissues with collagen sponges to develop novel tissue complexes ex vivo in TECs placed in the abdominal wall [120].

In cases where bone regeneration is required, the use of natural polymers or alloplastic materials as scaffolds and the desired perfusion offered by the in vivo regeneration process may be a novel solution to many clinical problems that require prolonged operations with significant patient morbidity from the use of extensive autologous tissues. ADSCs can actively participate in this process because of their differentiation potential into osteoblasts or chondrocytes, but this depends on the characteristics of the scaffold and the corresponding molecular signaling [121]. However, there is limited evidence for the impact of exosomes in this setting and more research is required.

The comparison of exosomes from ADSCs and human fibroblasts (HFFs) in skin flap survival revealed that different exosomes are secreted by each cell population with NGS analysis detecting the upregulation of miR-760 and downregulation of miR-423-3p in ADSC-exos. As a result, both ADSCs and ADSC-exos outcompeted HFFs and their exosomes, inducing increased angiogenesis and flap survival, as determined during microangiography. Furthermore, the effect on angiogenesis and flap survival was similar between ADSCs and ADSC-exos, suggesting that cell therapy is perhaps unnecessary during in vivo tissue engineering and acellular exosome therapies are rather advantageous and likely to have the possibility of being used in clinical studies sooner in the future [122]. However, data are limited, and further experimental studies are needed to substantiate this hypothesis.

The periosteum possesses active osteoprogenitor cells with osteoinductive potential that can be further enhanced when combined with bioactive scaffolds. Materials such as hydroxyapatite and bioactive glasses, a special type of ceramic, have been used to induce de novo osteogenesis in periosteal flaps [123]. This method is also described as neo-osseous flap prefabrication and it shows particular success in early experiments [124]. However, there have been no studies on the effectiveness of exosomes in bone regeneration with periosteal flaps in vivo and how they may possibly affect the survival and differentiation of osteogenic cells.

Composite 3D scaffolds can be used when tissue deficits are extensive and complex in order to restore the normal state. Examples are almost all cases involving the loss of cartilaginous external organs (nose, ear, larynx, trachea), the functional significance of which, in most cases, exists due to the complex structure and physiological characteristics of cartilaginous tissue. The ideal solution to this problem would be the use of composite, 3D-engineered biomaterials that would structurally mimic the normal organ and combine compatible materials with different cell types that would individually line each epithelial surface [125,126]. Although large and complex scaffolds are still in the experimental stage, clinical studies in patients with nasal cartilaginous defects showed excellent results. Complete cartilaginous structures had been developed in vitro from the patient’s own nasal chondrocytes and were safely used to restore the nasal anatomy. The scaffold material used in this study to grow the chondrocytes was Chondro-Gide, a porous bovine collagen I/III membrane [127].

A new field of interest in modern plastic surgery is lymphatics and lymphedema treatment. In addition to modern surgical techniques, technology has also made significant progress through materials and scaffolds that favor the growth of new lymph vessels in vivo or in combination with free lymph node flaps. BioBridge is a collagen nanofibrous scaffold that works synergistically with lymph node transfer to promote neolymphangiogenesis [128,129]. Exosomes in this field, although still at an early stage, seem to be able to participate through their cargoes, such as VEGF (CD63-VEGFC fusion protein), which are transferred to the endothelial cells of the lymphatics. In there, combined with sodium alginate hydrogels, they may eventually lead to the improvement of lymphedema [130].

## 8. Clinical Trials and Current Legislation Status of Exosomes for Clinical Purposes

There are currently several ongoing clinical trials that explore the therapeutic potential of ADSC-exos in diabetic wound healing by restoring fibroblast function, in obesity and metabolic disorders by modulating inflammation and immune response, as well as in COVID-19-associated pneumonia and hepatic fibrosis by suppressing inflammation and promoting tissue repair [36,37]. 

Although many of these trials are in the early preclinical stage, there are three that have been published. One Korean study focused on the effect of ADSC-exos on acne scars after CO_2_ laser treatment while the other two studies from China utilized allogeneic ADSC-exos to study the efficacy and the clinical safety of exosomes in diseases like Alzheimer’s or severe COVID-19 with positive results [131,132,133].

The regulatory landscape for exosome-based therapeutics is constantly evolving and both the FDA as well as the European Medicines Agency (EMA) are working on safety and efficacy guidelines. Although the FDA has not actually released any guidelines specific to exosomes, the regulatory process is comparable to that for other biologics and cell-based therapies, demanding rigorous preclinical and clinical testing.

On the other hand, the EMA has released guidelines which include exosome-based therapies, emphasizing the good characterization of exosome products, good manufacturing practices, and clinical testing requirements to ensure that exosome therapies are safe, effective, and consistently manufactured across different batches [134].

Up to date, there is only one phase I/II clinical trial (NCT06279741), the EVENEW study, that has assessed the safety and efficacy of the intratracheal administration of EXOB-001, a novel EV-based drug, in preventing Bronchopulmonary Dysplasia (BPD) in preterm newborns. This is the first MSC-derived interventional study that EMA has approved. 

## 9. Conclusions and Future Perspectives

The undiscovered value of adipose stem cell exosomes originally in oncology and more recently in regenerative medicine continues to be revealed through the decisive collaboration between the researchers and clinicians of different specialties [135]. They key areas of ADSC-exo applications include the enhancement of wound healing and skin regeneration by promoting collagen synthesis, fibroblast proliferation, and angiogenesis, suitable for chronic wound management and burn injuries, as well as cartilage and bone regeneration by promoting chondrogenic differentiation and cartilage repair suitable for osteoarthritis and the healing of bone fractures. ADSC-exos can also support liver and kidney regeneration, promoting proliferation and reducing fibrosis in chronic liver disease and kidney injuries as well as exhibiting immunomodulatory roles suitable for the treatment of inflammatory and autoimmune diseases. However, more novel applications include cardiovascular repair in the context of myocardial infarction and neurological conditions including stroke recovery and neurodegenerative diseases as well as cosmetic and dermatological approaches.

Although positive results have been achieved in preclinical studies that reveal the well-tolerated and safe results of exosomes, the clinical application of ADSC-exos still faces challenges in specificity, separation, and isolation methods as well as in safety protocols and in establishing their pharmacokinetic and pharmacodynamic characteristics. 

The future perspectives of the ADSC-exo field involve the development of standardized protocols for the isolation, characterization, and quantification of ADSC-exos to enable consistency in quality and efficacy as well as scaling up production for clinical use. Targeted delivery systems should be explored either through the engineering of exosomes with surface modifications to enhance specific targeting or through an effective combination with biomaterials or nanoparticles to improve their stability and delivery efficiency. In addition, randomized large-scale clinical trials need to be conducted in order to establish their safety and efficacy as well as guided regulatory processes to achieve approvals for exosome-based therapies. Further mechanistic studies are also required to elucidate the underlying molecular mechanisms and investigate the interaction between exosomes and recipient cells to enhance their therapeutic potential.

In conclusion, the field of exosomes is broad and promising for a wide range of clinical applications in tissue regeneration but also bears many challenges. Rigorously standardized research and clinical investigations are imperative to elucidate the full spectrum of their diagnostic and therapeutic potential and translate these advances into effective, widely available therapies.

## Figures and Tables

**Table 1:** Advantages and disadvantages of different surgical techniques for obtaining adipose tissue.

	Advantages	Disadvantages
**Liposuction with a vacuum machine**	Short time for the collection of larger fat volume Small scar	High negative pressure possibly leads to fat cell destruction Required specific equipment—vacuum suction
**Liposuction with vacuum syringe—Coleman Technique**	Minimal trauma to fat cells Small scar Adequate amount of fat	Laborious technique More time for a similar amount of fat tissue compared to vacuum suction
**Surgical resection**	The structure and viability of the harvested tissue maintained	Significant scar—the disruption of donor area architecture Reduced tissue plasticity

**Table 2:** Potential therapeutic effects of ADSC-exos.

Origin of Exosomes	Cargo	Target Cells	Mechanism of Action	Effects	Reference
ADSC-EVs	miR-486-5p	HDF and HMECs	Neoangiogenesis	Improved healing	[57]
ADSC-exos	miR-125a, miR-31	HUVECs	The enhancement of pro-angiogenic activity by targeting FIH1 in endothelial cells	Angiogenesis	[58,59]
ADSC-exos	lncRNA MALAT1	HDF/HaCaT	HDF proliferation and migration, Wnt/β-catenin activation through miR-124 binding, and the inhibition of oxidative stress	Improved healing	[60,61]
ADSC-exos	-	EPCs	Nrf2 expression and the inhibition of oxidative stress	Neoangiogenesis/improved healing in the hyperglycemic state	[62]
ADSC-exos	-	HDF	Increase in TGF-β3, collagen III, and MMP-3 through the ERK/MAPK pathway	Improved healing	[65]
ADSC-exos	miR-21	HaCaT	The activation of keratinocyte proliferation and induction of MMP-9	Improved healing in tissue deficits	[67,68]
ADSC-exos	CD9, CD63, AGO2 (as surface molecules)	HDF	The expression of VEGF-A, FGF-2, HGF, and PDGF-BB, and the conversion of fibroblasts to myofibroblasts	Improved healing	[69]
ADSC-EVs	miR-23a, miR-23b, miR-let7, miR-24, miR-125b miR-16	Macrophages	Proangiogenesis, M2 macrophage polarity, and the downregulation of MYD88/NF-κB, IL-6/TNF-α, and IKK-α	Muscle regeneration	[72,73]
ADSC-exos	miR-145, miR-221	Periosteal cells and ADSCs	Guiding cells to differentiate, KGN-induced ADSC-exos promote ADSCs into chondrocytes	Chondrogenesis	[74,78]
ADSC-exos	miR-130a-3p	ADSCs	Increase in Runx2 and ALP and the osteogenic differentiation of ADSCs via SIRT7/Wnt/β-catenin	Osteogenesis	[75,76]
AT-EVs	miR-450a-5p	ADSCs	The differentiation of ADSCs to mature adipose tissue through WISP2 inhibition	Lipogenesis	[81]
ADSC-exos	-	Schwann cells	The overexpression of Ccnd1, Ccna2, Rac1, Cthrc1, and MMP-9	Neural regeneration	[82]
ADSC-exos	miR-21	HUVECs	The promotion of endothelial cells towards angiogenesis	Angiogenesis	[85]
MSC-exos	miR-1, miR-133, miR-206, miR-494	Muscle cells	Increase in VEGF and IL-6	Muscle regeneration and angiogenesis	[86]
